# Contact Lens Practices and Knowledge of Complications and its Association With Refractive Error in Saudi Arabia

**DOI:** 10.7759/cureus.12786

**Published:** 2021-01-19

**Authors:** Othman Alzahrani, Fayez A Alshehri, Abdulrahman O Alali, Omar H Alzahrani, Zaid A Alzahrani, Abdulrahman AlZahrani, Abdulrahman A Almazrou

**Affiliations:** 1 Medicine, Imam Muhammad ibn Saud Islamic University, Riyadh, SAU; 2 Urology, King Saud bin Abdulaziz University for Health Sciences, Jeddah, SAU; 3 Ophthalmology, Imam Muhammad ibn Saud Islamic University, Riyadh, SAU

**Keywords:** contact lenses, complications, practices, refractive error, saudi arabia

## Abstract

Introduction

For optical, cosmetic, or therapeutic purposes, contact lenses (CLs) are temporary prostheses positioned on the eye. CLs do not only improve the quality of life by correcting, but also providing a better appearance and less activity restriction. Patients’ failure to comply with the hygienic practices prescribed in using CLs is often considered an important risk factor for eye complications and it is not often clarified to CLs consumers at the time of dispensation.

Aim

The objective of this study was to assess the knowledge and practices towards the use of CLs in a sample of the Saudi population in Riyadh.

Methods

A cross-sectional study was performed using an online questionnaire among adult residents in Riyadh, Saudi Arabia from April to May of 2020. The statistical analysis was performed using R v 3.6.2 (R Foundation for Statistical Computing, Vienna, Austria). Ordinal (Likert-scale) variables were summarized using mean ± standard deviation, or median and interquartile range (IQR) as needed.

Results

The majority of current users of CLs had no sight problem (P < 0.001). Using CLs was thought to be harmful in non-users (12.4%) versus CL users (2.93%) (P < 0.001). The main reason for using CLs in all groups was the emulation of others. Dryness was a well-known complication in current users (P < 0.05) and evening discomfort was a well-known one in previous users (P < 0.05). Social circle was the main source of information in 40% of users. High monthly income has a significant association with knowledge regarding the complications of CLs compared with low income (B = 0.94, P < 0.05). Education has a major effect on the users’ knowledge regarding the increase of refractive error and infection by CLs (P < 0.05).

Conclusions

Knowledge and practice were less than desired among CLs users. Many use CLs without prescription and solely for cosmetic purposes. More education is needed to lessen eye complications among CLs users.

## Introduction

Contact lenses (CLs) are commonly used for refractive error correction and cosmetic purposes. Patients with refractive error can replace eyeglasses with CLs which help them to improve the quality of life and to provide flexibility during activities, which is a more preferable option in younger age groups [1,2].

Because CLs are readily available over the counter without prescription, it is likely that the community lacks knowledge on the safety and risks associated with their uses. The educational level regarding the safe use of CLs should be uplifted to the appropriate use, hygiene techniques, and possible consequences that may result from the misuse of CLs for all age groups and even for health staff [3-5]. Unfortunately, the failure to adhere to the medical instructions may result in unforeseen complications which can be mild as conjunctival hyperemia, discomfort, and papillae formation requiring medical observation and simple symptomatic treatment. Moreover, the most serious complication is blindness which evolves from microbial keratitis and endophthalmitis [6].

In 2015, the Centers for Disease Control (CDC) reported the number and prevalence of adult CLs users in the United States and identified the population at risk for contact-related eye infections. Exposing lenses to water, sleeping with lenses, reusing sterilization solutions, and not adhering to replacement schedules were the most common risk factors for severe complications [7].

Several studies showed that adolescents and young adults were more prone to develop CLs complications than older adults [8]. The objectives of this study were to determine the extent of knowledge and practice for CLs users in Riyadh and to assess their knowledge on some of the complications that may exist as consequences of CLs.

## Materials and methods

A cross-sectional study was performed using an online survey questionnaire hosted on the cloud-based software SurveyMonkey (SVMK Inc, San Mateo, CA, USA) was sent to adult residents in Riyadh from April to May 2020. This survey was approved by the Ethical and Research Committee of our institute. Due to the coronavirus disease 2019 (COVID-19) crisis and as a part of the social distancing measures, written consent was waived by the Institutional Review Board (IRB) and it was replaced by a preceding question prior to the survey questionnaire confirming the agreement of participants to enter the study.

The questionnaire used in this survey is a validated and modified version of previously published research [9,10]. Self-reported answers to the questionnaire had been used for data collection. Inclusion criteria include people who used CLs for refractive error or cosmetic purposes in addition to Saudi citizens who never used CLs. Those who are less than 18 years old or residing outside of Riyadh were excluded from the study. The Arabic version of the questionnaire's validity and reliability was confirmed by a pilot sample. The questionnaire was designed to assess the knowledge and practices towards CLs uses and their complications. The survey form included demographic characteristics (gender, age group, educational level, and monthly income), frequency of visiting ophthalmologists, problems of sight that require CLs, duration of using eyeglasses, CLs usage of current or previous or non-user, and reasons for using/avoiding CLs. In addition, it included questions related to knowledge, practice, type and complications of CLs, as well as duration and frequency of wearing CLs. Other questions were about how participants started using CLs, factors influencing CLs use, knowledge regarding adverse effects of CLs, frequency of adverse effects, compliance with safety measures related to CLs use, concerns related to CLs use among non-users, and finally, sources of information about CLs, their uses and complications. The participants were instructed to give truthful and honest answers. The responses to the questionnaire were and will be kept confidential. A total of 1,356 people (18 years and older) living in Riyadh participated in the survey.

Statistical analysis was performed using R v 3.6.2 (R Foundation for Statistical Computing, Vienna, Austria). Variables were summarized using numbers and percentages. Ordinal (Likert-scale) variables were summarized using mean ± standard deviation or median and interquartile range (IQR) as needed. Adverse events of CLs use were graded from 1 (never) to 5 (always). Chi-square test of independence was applied to assess the association among categorical variables. Linear regression was used to evaluate the factors associated with knowledge scores. Estimates (B) and standard errors were calculated; 95% confidence intervals were constructed using these estimates. Confidence intervals that do not include 0 were considered statistically significant. Hypothesis testing was performed at 5% level of significance. P values <0.05 were considered statistically significant. Factors associated with knowledge regarding safety measures in users of CLs were analyzed by using a cumulative knowledge score which was calculated for each participant as follows: participants were awarded 1 point if they chose “yes”, the correct answer otherwise they were given zero. The total points were then calculated for each participant. Independent variables included sex, age, type of CLs, use of CLs (previous vs. current), education, and average monthly income.

## Results

The questionnaire of the study was responded to by 1356 participants with gender distribution of 405 males (29.9%) and 950 females (70.1%). Participants aged 18 - 30 years represented 65.1% of the study sample. The majority of the respondents (67.8%) completed university education with monthly income ranging from < 5000 SAR (51.8%) to > 15000 SAR (16.2%). 68.3% of the respondents visited ophthalmologists only when needed. Interestingly, 23.2% of the participants mentioned that they never visited their ophthalmologist. Almost half of the respondents (51.4%) had sight problems that required the use of CLs. Slightly less than half of the respondents (44.5%) never used eyeglasses. Among those who used eyeglasses, the duration was variable. Those who used eyeglasses for < 1 year represented 10.7% of the sample while those who used eyeglasses for 1 - 5 years and 5 - 10 years represented 15.8% and 14.2% of the study sample, respectively. Respondents who used glasses for > 10 years represented 14.8%. On the other hand, The participants who never used CLs represented 52.4%. Current and previous users of CLs represented 15.1% and 32.5%, respectively (Table 1).

**Table 1:** Demographic characteristics of the study sample

	N (%)	Valid N
Gender:		1356
Female	950 (70.1%)	
Male	405 (29.9%)	
Age:		1356
18-30	882 (65.1%)	
31-40	208 (15.4%)	
41-50	162 (12.0%)	
51-60	91 (6.72%)	
above 60	12 (0.89%)	
Education:		1356
Primary	59 (4.35%)	
High school	377 (27.8%)	
Bachelor degree	920 (67.8%)	
Monthly income:		1356
< 5000 SAR	702 (51.8%)	
5000 - 15000 SAR	434 (32.0%)	
> 15000 SAR	219 (16.2%)	
Frequency of visiting ophthalmologist:		1356
Never	314 (23.2%)	
When needed	926 (68.3%)	
Regularly	69 (5.09%)	
Other	47 (3.47%)	
Sight problem that requires contact lenses:		1356
No	659 (48.6%)	
Yes	697 (51.4%)	
Duration of using glasses:		1356
Never	603 (44.5%)	
Less than a year	145 (10.7%)	
1 to 5 years	214 (15.8%)	
5 to 10 years	193 (14.2%)	
More than 10 years	201 (14.8%)	
Contact lens use:		1356
Never	710 (52.4%)	
Previous user	441 (32.5%)	
Current user	205 (15.1%)	

The results showed that among all the people who responded to the survey with no sight problem, the majority are current CLs users (P < 0.001). Responses also indicated that 19.9% of those who never used CLs found the use of glass comfortable (P < 0.001) and that 40.6% of them had no interest in using CLs. The percentage of respondents that found the use of CLs uncomfortable was higher in those who never used CLs (17.9%) and in previous users (19.5%) compared to current users (8.78%). The prevalence of medical problems was not statistically significantly different among the three groups (P value= 0.652). Non-users were more likely to report their impression that CLs were harmful with 12.4% versus 2.93% (P value < 0.001) (Table 2).

**Table 2:** Reasons for using or avoiding contact lenses (CLs)

	Never	Previous user	Current user	P
	N=710	N=441	N=205	
No sight problem:	46 (6.48%)	134 (30.4%)	140 (68.3%)	<0.001
Sight problem:	32 (4.51%)	24 (5.44%)	12 (5.85%)	0.652
Using glasses is comfortable:	141 (19.9%)	70 (15.9%)	14 (6.83%)	<0.001
Using CL is harmful to the eye:	88 (12.4%)	38 (8.62%)	6 (2.93%)	<0.001
My ophthalmologist does not recommend CLs:	60 (8.45%)	17 (3.85%)	4 (1.95%)	<0.001
CLs are expensive:	26 (3.66%)	27 (6.12%)	7 (3.41%)	0.107
No interest:	288 (40.6%)	58 (13.2%)	1 (0.49%)	<0.001
Using CLs is difficult:	119 (16.8%)	44 (9.98%)	8 (3.90%)	<0.001
Using CLs is uncomfortable:	127 (17.9%)	86 (19.5%)	18 (8.78%)	0.002
Other:	142 (20.0%)	93 (21.1%)	34 (16.6%)	0.405
% for each response was calculated from the total number of respondents Statistical analysis was performed using Chi-square test of independence				

**Figure 1:**
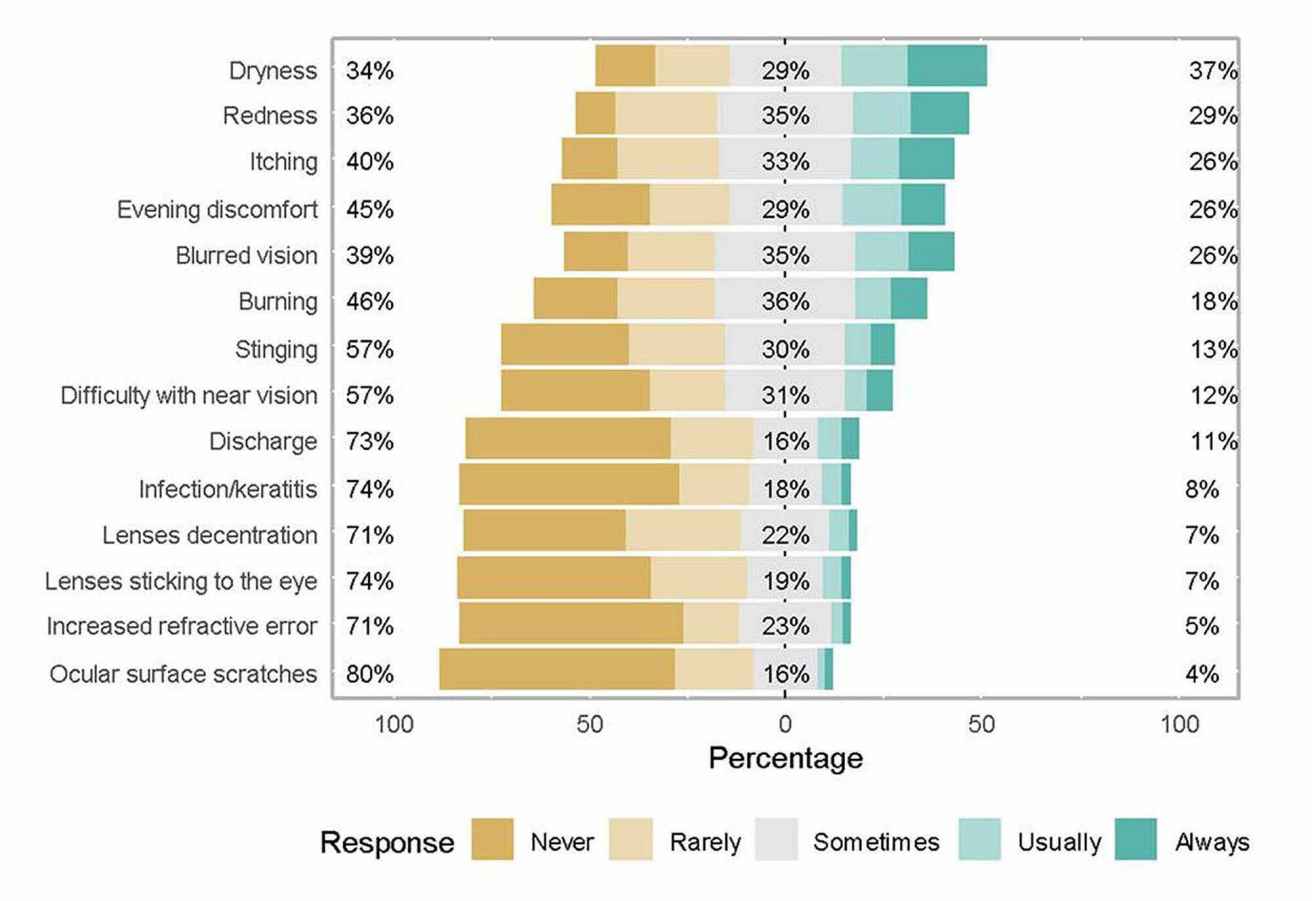
Knowledge about the adverse effects of contact lenses (CLs)

No statistically significant difference was observed in the knowledge of the various adverse effects of CLs except for dryness which was a well-known complication in current users (P < 0.05) and evening discomfort in previous users (P < 0.05). The results showed that dryness (37%) and redness (29%) were the most known adverse effects. The least common adverse effects that are known by respondents included increased refractive error (5%) and ocular surface scratches (4%) (Figure 1).

**Figure 2:**
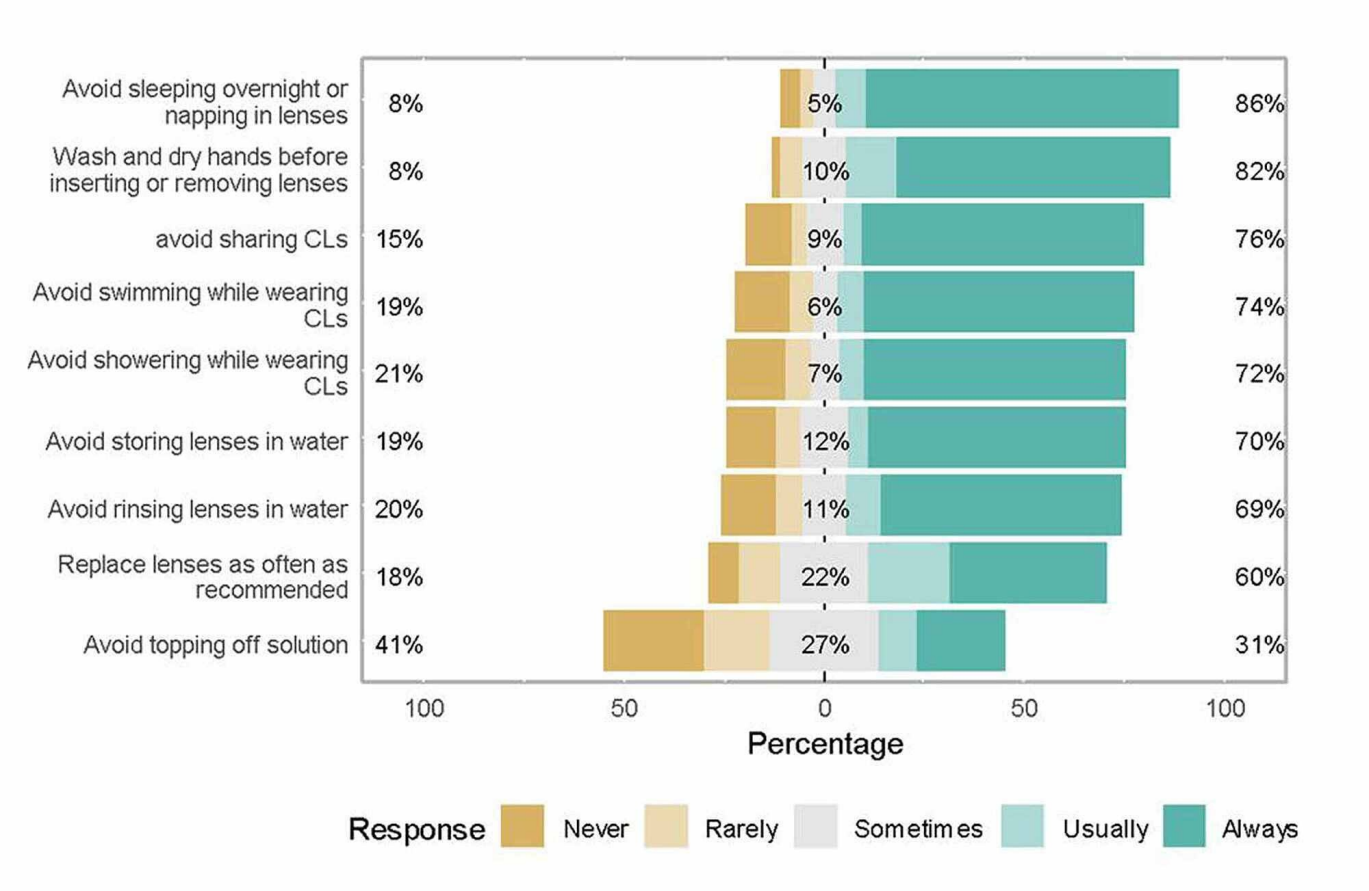
Knowledge about safety measures in users of contact lenses (CLs)

The most common safety practices followed by the respondents included avoiding sleeping overnight or napping with lenses (86%), washing and drying hands before inserting or removing lenses (82%), and avoiding CLs sharing (76%). The least common safety practices performed by respondents were “avoid topping off solution” and replacing lenses as often as recommended. The results showed that knowledge regarding safety measures related to CLs use was significantly higher in current users compared to previous users. The current users were more likely to choose “wash hands before inserting or removing lenses” compared to the previous users (92% vs. 81.2%, P value < 0.05). The current users were also more likely to choose “replace lenses as recommended” compared to the previous users (66% vs. 50.2%, P value < 0.05) and they were also more likely to choose “storing or rinsing lenses in water”. Knowledge regarding other practices was not statistically significant between both groups (Figure 2).

For factors associated with knowledge regarding complications of CLs, a cumulative knowledge score was calculated for each participant as follows: the participant was awarded 1 point if he/she chose “yes”, the correct answer; otherwise, he/she was given zero. The total points were then calculated for each participant. Linear regression was used to assess the factors associated with knowledge score. Independent variables included sex, age, type of CLs, use of CLs (previous vs. current), education, and average monthly income. A maximum score of 14 was possible.

The results showed that gender was not associated with knowledge about complications of CLs (B = -0.1, P > 0.05). Age showed a statistically significant association with knowledge about complications of CLs. The average knowledge score was lower by 1.1 and 2.26 points among participants aged 31 to 40 years (P = 0.001) and those aged 41 - 50 years (P < 0.001) compared to participants < 30 years. Education did not show a statistically significant association with knowledge about complications of CLs. Monthly income showed a statistically significant association with knowledge about complications of CLs; the participants who earned > 15000 SAR showed higher knowledge compared to those earning < 5000 SAR (B = 0.94, P < 0.05). Current use of CLs was not associated with better knowledge about complications of CLs (Table 3).

**Table 3:** Factors associated with knowledge about complications of contact lenses (CLs)

Predictors	Estimates	CI	p
(Intercept)	3.10	1.26 – 4.95	0.001
Gender: Female	Ref		
Gender: Male	-0.10	-0.93 – 0.74	0.822
Age: < 30 years	Ref		
Age: 31-40	-1.10	-1.77 – -0.43	0.001
Age: 41-50	-2.26	-3.11 – -1.41	<0.001
Age: 51-60	-1.41	-2.95 – 0.13	0.073
Age: above 60	-2.89	-8.07 – 2.29	0.273
Education: Primary	Ref		
Education: High school	0.75	-1.16 – 2.66	0.438
Education: Bachelor degree	0.79	-1.08 – 2.66	0.406
Monthly income: < 5000 SAR	Ref		
Monthly income: 5000-15000 SAR	0.40	-0.22 – 1.02	0.210
Monthly income: >15000 SAR	0.94	0.11 – 1.77	0.026
Contact lens use: Previous user	Ref		
Contact lens use: Current user	-0.21	-0.73 – 0.32	0.433

## Discussion

CLs are commonly used for refractive error correction as well as therapeutic and cosmetics purposes. Our survey sheds light upon public practices regarding CLs uses and complications among patients with or without refractive error in the Riyadh population. Half of the respondents had sight problems that required the use of CLs. Those who never used CLs represented 52.4% of the study sample. Third of the responders used CLs for cosmetic reasons. In a Saudi study published by Alobaidan et al., nearly two-thirds of the participants used CLs for cosmetic purposes [9]. In another study conducted by Abahussin et al. among Saudi university students, a similar segment of CLs users applied them for cosmetic purposes [10].

In our study, 23.2% mentioned that they had never visited an ophthalmologist. The majority of the respondents visited ophthalmologists only when needed (68.3%). Similarly, Alobaidan et al. found that huge numbers of CLs users had visited eye care professionals for complication management indicating that they might have faced a sight-threatening problem [9]. Interestingly, using eyeglasses increased the likelihood to use CLs. In contrast, the participants who never used eyeglasses were less likely to use CLs. This indicates that for Saudis, CLs are used more as complementary to eyeglasses rather than as an alternative.

19.9% of those who never used CLs found that the use of eyeglasses was more comfortable, and 40.6% of them had no interest in using CLs. In other words, the rate of respondents who found the use of CLs uncomfortable was higher in those who never used CLs. Non-users of CLs were mainly concerned with whether cataract development was associated with the use of CLs or not. Şengör et al. remarked that, for people who did not prefer CLs, the main reason behind avoiding CLs use was the belief that they were difficult, and the main concern was the possibility of eye infection during CLs use [11].

While it is expected that a large portion of people who responded to the survey have never used CLs to show a less-than-optimal level of knowledge, the results demonstrate that non-users of CLs with refractory error may have unnecessary apprehensions that prevent them from correcting their vision through the use of CLs. Many factors were attracting people to use CLs. However, emulation of others was more prevalent in all groups of CLs users. Şengör et al. noted that CLs were considered optically and esthetically superior to glasses [11]. This implies that while non-users may prefer the use of CLs over eyeglasses, an excessive fear of complications and risks prevent them from doing so, which can be mitigated by making them more informed. 

Regarding compliance with the safety measures related to the CLs use, the most common safety practice followed by the respondents was avoiding sleeping overnight or napping with lenses and the least common safety practice performed by the respondents was “avoiding topping off solution”. These findings are opposite to Cope et al. who noted that around six of seven lens wearers reported at least one behavior that put them at risk for serious eye-related contact-lens infection, most frequently, sleeping or napping with lenses [12]. Wu et al. assessed non-compliant habits of CLs users and listed hand hygiene, poor lens care, and failure to remember the follow-up appointments as the key issues [13]. In our study, while users of CLs tended to display relatively better knowledge regarding correct use and tended to be aware of safety practices, they still had poor knowledge of possible complications of CL misuse, which might affect their decision to use CLs for corrective or cosmetic reasons.

We found out that around 40% of the respondents took the information about CLs from the social circle. Şengör T et al. noted that only 56% of the participants of the study received information from their ophthalmologists regarding how to use CLs [11]. In our study, the proportion of participants who chose ophthalmologists and opticians as a source of information was only 20.7% of Cls users.

Alobaidan et al. showed that knowledge and practice among prescribed CLs users were significantly better compared with CLs buyers 'over the counter.' [9]. Restricted directions and advice by vendors to CLs users might be responsible for poor knowledge and practices among the Saudi population. It seems that there is an urgent need for decision-makers to intervene in the provision of CLs to provide them with a health promotion package in Saudi Arabia for standard use and care of CLs. Ayesha et al. found that the prompt-access CLs sold at night or on the flea market pose a major problem and it could cause further contact-lens ulcer [14].

We remarked that dryness and redness were the most commonly known adverse effects among CLs users. On the other hand, the least common adverse effects known by respondents included increased refractive error and ocular surface scratches. Alipour et al. showed that CLs discomfort incidence varied in patients from 23% to 94% [15].

We noted the association of a better level of knowledge about safety measures and the complications related to CLs use in young adults compared to those older than 30 years of age. Alobaidan et al. had a similar finding that younger people, being more educated, having a wider scope of accessing knowledge, and preferring practice norms for CL use, were at less risk of complications [9]. The level of education is reflected in the knowledge about safety measures; the average knowledge was higher in participants with a bachelor’s degree compared to those with high school and primary education. High monthly income showed higher knowledge about the complications of CLs than low monthly income.

In our study, the gender of participants was not correlated with the level of knowledge about safety measures or complications of CLs. We noted that 70.1% of the CL users in our study were female, which is indicative of a gender bias in CLs use, yet is still less than a previous study conducted on a smaller sample in Saudi Arabia which found CLs users to be 92% female [9]. Similar findings of higher CL use among females have also been demonstrated in other countries, with women being 40% more likely to use contacts than men [16]. This implies that while larger samples may demonstrate a closer gap in gender, women are still more likely to use CLs than men in Saudi Arabia. In addition, the majority of participants were aged 18 - 30 years, which demonstrated that imparting knowledge about preferred CL usage should be age and gender-specific in Saudi Arabia for better practices. Current users of CLs have better knowledge about safety practices than previous or non-users of CLs. On the other hand, we noticed that the current users of CLs lack knowledge about the complications of CLs.

There are limitations to these findings which are mainly the cross-sectional design and the convivence sampling technique. Future studies with a different design may yield new findings.

## Conclusions

Knowledge and practice were less than desired among CLs users. Many of them used contact lenses for aesthetic reasons and procured them without prescription. There is a need for CLs consumers to have more knowledge about CLs care and complications, which should be provided by all CLs providers. Consequently, the prevalence of eye complications will be lessened among CLs wearers.
